# Two Cases of Sporadic Cholera

**Published:** 1873-08-15

**Authors:** F. H. Davis


### TWO CASES OF SPORADIC CHOLERA.

REPORT READ BEFORE THE CHICAGO SOCIETY OF PHYSICIANS AND SURGEONS, MONDAY, JULY 28, BY F. H. DAVIS, M.D.

CASE I.—On Tuesday, July 15th, I was sent for in great haste, about noon, to see a boy at 389 Fourth avenue. I found the patient, a lad about fifteen years old, had been out on a picnic the day previous, and had, according to his own statement, spent a considerable time bathing in the lake, and had drank excessively of lemonade and ice-water. On his return home, late in the evening, he retired to bed. About 2 o'clock in the morning he was taken with violent vomiting, purging, and cramps. Some physician, I did not learn who, was called in, and prescribed some powders, which, however, were given only once or twice and then immediately ejected. The matter passed from the bowels, as well as the ejections from the stomach, consisted of simple, clear, watery mucous, with some white, curdy-looking particles intermixed.

When I saw him, at noon, the extremities were cold; pulse barely perceptible at the wrist; lips and nails blue; eyes and cheeks sunken; the stage of collapse evidently about commencing. The cramps continued severe, but the discharges had almost entirely ceased, apparently from simple exhaustion.

The friends of the boy belonged to the lowest and most ignorant class of Irish; and it was evidently hopeless to try to get any directions carried out efficiently or perseveringly. The dejections were allowed to stand unemptied, to soil the bedding, etc. Under the circumstances, I entertained no hopes of accomplishing anything in the way of treatment.

Having to pass the house again about 2 P.M., in company with my father, I requested him to stop and see the case with me. The boy had continued to fail rapidly, and had passed into complete collapse. The hands and fingers presented a blue, shrunken, shrivelled appearance. No perceptible pulse; breathing slow and labored; heart-sounds weak. Death ensued in about half an hour after our last visit.

CASE II.—The previous Friday, July 11th, Dr. N. S. Davis was called to an exactly similar case, on the lower end of Catherine street, near the river. He called, expecting to meet in consultation another physician, who had seen the case previously. Through the stupidity or intentional deception of the friends, however, the attending physician had been discharged before his arrival. He found a boy of fourteen, who had been taken with vomiting and purging at 1 P.M. of previous day. The active symptoms were then abated, but the stage of collapse was just setting in. The boy had been working hard in the hot sun the day previous; had drank excessively of ice-water, and, as a consequence, had sweat profusely. My father left directions for treatment, and agreed that if the boy lived through the night I should visit him early the next morning. On my arrival the following morning, however, he was dying, in complete collapse, the same as the previous case.

Both these cases occurred in low, filthy, undrained districts of the city, where the ditches are piled up full of garbage and filth, and many of the lots and back alleys covered with green, stagnant water.

Among the poorer classes inhabiting these unhealthy districts of our city, there always occurs, every summer, along with numerous milder cases of cholera-morbus, a few cases similar to these. Many physicians would class them as sporadic cases of genuine cholera, not becoming epidemic simply because the atmospheric conditions do not happen to be favorable to its spread and propagation.

Those, however, who hold to the doctrine that cholera infection must necessarily be introduced from abroad — that, originating in Asia, it travels from one city to another, along the channels of commerce, etc. — the advocates of this theory, of course, recognize true cholera only in its epidemic form, and class all scattering cases such as the above as severe cholera-morbus.

Most of our older physicians, who have had most experience with cholera in former epidemics, have been predicting a visitation from it here during the present summer, drawing their conclusions partially from the fact of its prevalence in other cities in various parts of the country, and partially from the character of the atmospheric conditions which the season has already manifested.

Even those who have no faith in the direct transmission of the disease from place to place, by means of a *specific* poison, yet do not pretend to deny that its epidemic prevalence in one part of the country is likely to be followed by more or less of an epidemic in all the other districts, in succession. There are evidently some peculiar atmospheric conditions or changes, the natures of which we cannot yet fully appreciate, but which, sweeping over the country, or even from continent to continent, with almost the same regularity as the wind and rain storms, so act on the system as to weaken its vitality and powers of resistance to morbid or unhealthful impressions. Under these circumstances, then, the decaying animal or vegetable matters, the stagnant pools, etc., form the hot-beds for the dissemination of epidemics — not because there is any *specific* poison emenating from them at one time more than another, but simply because, in a reduced, debilitated state of the system, they obtain the mastery, where, in the ordinary vigor or health, the constitutional strength would have been sufficient to throw off or overcome their influence.

In the same way, the sporadic cases that occur at irregular intervals during the summer months can invariably be traced to exposure to some of these poisonous influences, while the system was weakened from the effects of some excesses committed by the individual.

The cholera epidemic which has been prevailing in various parts of the country during the present season, seems to have pursued a very irregular, erratic course, passing by entirely some of the larger cities, which had suffered most in former epidemics, and turning aside to attack, with the greatest virulence, the outlying suburbs or surrounding villages, and many of the smaller, less thickly populated cities.

The reason for this we think is evident. Warned by sad experience in former years, all of our larger cities have been placed under efficient sanitary control. Especial pains have been taken to cleanse all the streets and alleys, and to remove promptly all filth and garbage. These timely measures, together with the modern improvements, in the way of perfected underground drainage, clean, paved streets, and an abundant supply of pure water, renders most of our larger cities practically impregnable to the cholera scourge. The predisposing atmospheric influences may be present, but they have no power to generate the disease without the more direct, exciting causes. Isolated cases will occur here and there; *but they do not generate others;* the disease gains no foothold, and quickly dies out.

In many of the smaller places, however, which have been suffering most during the present season from cholera, there has been no attempt to place the streets in a proper sanitary condition. There have been no sewers; imperfect drainage; no public water supply, dependence having to be placed upon wells and cisterns, many of them filled only with impure surface water, etc.

If properly investigated, the spread and propagation of cholera can, we believe, be traced, in every identical instance, to some of these local, exciting causes, which proper, intelligent care and forethought might have removed.

Some facts lately evolved seem rather to indicate that a deficiency of *ozone* and *electricity* in the atmosphere has some influence in favoring the development of cholera, and perhaps other epidemic diseases.

As far as the influence of these agents is known and can be studied, they seem to impart a *tonic*, *exhilarating* element to the atmosphere, any considerable deficiency in which is almost immediately perceptible in its depressing, enervating effect upon the organism.

Further and more extended observation and experiment are wanted to *prove* the connection which seems to exist between these influences and the epidemic diseases. We feel confident, however, that it is in this direction, if at all, that we are to look for success in the unraveling of the mystery which involves the origin and spread of cholera.

